# Multiple Renal Infarctions in Spontaneous Double Renal Artery Dissection: A Case Report

**DOI:** 10.3390/jcm13237307

**Published:** 2024-12-01

**Authors:** Gaetano Ferrara, Michelangelo Nasuto, Francesco Napolitano, Giovanni Ciccarese, Filippo Aucella

**Affiliations:** 1 Nephrology and Dialysis Unit, IRCCS Casa Sollievo Della Sofferenza Hospital, 71013 San Giovanni Rotondo, Italy; francesco.napolitano@unifg.it (F.N.); f.aucella@operapadrepio.it (F.A.); 2 Department of Radiological Sciences, Fondazione IRCCS Casa Sollievo Della Sofferenza, 71013 San Giovanni Rotondo, Italy; michelangelo.nasuto@gmail.com (M.N.); gcicca@yahoo.it (G.C.)

**Keywords:** renal artery infarctions, spontaneous renal artery dissection, renal imaging, interventional radiology, abdominal flank pain, case report

## Abstract

**Background:** As spontaneous renal artery dissection (SRAD) is a rare cause of abdominal pain, bilateral dissection is an extremely rare event. Only approximately two hundred cases of SRAD have been reported in the literature. The diagnosis is often delayed due to the rarity of the disease and non-specific clinical presentations such as flank pain, hypertension, fever, nausea, vomiting, and hematuria, which can be often misdiagnosed as a genito-urinary infection or gastrointestinal or bowel disease. Before 1980, the diagnosis of SRAD was mostly confirmed via autopsy or, rarely, via angiography. At present, the diagnosis is made using advanced imaging approaches, including computed tomography angiography (CTA) and magnetic resonance angiography (MRA), with a higher number of incidentally diagnosed SRADs. **Methods:** we performed laboratory tests and radiological examinations (computed abdominal tomography and multiplanar reconstruction) that revealed multiple infarctions and ischemic areas with hypoperfusion in the upper middle third of the left kidney and in a large part of middle and lower areas of the right kidney; the left renal artery exhibited increased intimal thickening and arteritis. **Results:** The multiplanar reconstruction revealed bilateral renal artery dissection and multiple arterial infarctions disseminated throughout both kidneys. After a clinical follow-up and hypertension retargeting, the patient was discharged with dual antiplatelet therapy and ACE inhibitor drugs. No lipid-lowering therapy was needed. **Conclusions:** Spontaneous renal artery dissection (SRAD) is a rare clinical event that typically presents with acute low-back or flank pain, hypertension, fever, hematuria, and acute renal failure. The condition could be misdiagnosed or receive a delayed diagnosis due to its relative rarity and non-specific presentation. The gold standard is enhanced computed tomography (CT) scans, and if the diagnosis is positive, vascular multiplanar reconstruction is generally suggested, as it can display lesions more clearly. Over 300 cases have been reported since the first characterization of SRAD; however, to date, a consensus has not been reached on the most appropriate treatment. Conservative therapy, open surgery, and intravascular intervention have been reported as treatments for SRAD.

## 1. Introduction

If isolated dissection of the renal artery is a rare event, bilateral dissection with multiple bilateral infarctions is an extremely rare event.

In fact, the natural history of this condition is poorly understood, with both benign and malignant outcomes having been described. Furthermore, the reported associated mortality rate is also variable. A meta-analysis of case reports over a 50-year period reported a 1% mortality rate, while another study reported a 17% mortality rate over 6 years [1].

## 2. Case Description

We report the case of a 49-year-old man who was referred to our Emergency Room with acute abdominal flank lower back pain, uncontrolled hypertension, headache, and malaise. These symptoms were intermittent and did not vary when the patient was standing. He declared that this had been occurring for the past two days prior to admission. He had taken paracetamol and non-steroid anti-inflammatory drugs (NSAIDs) at home, with partial amelioration in the symptoms.

His medical record was notable in that his parents had a history of arterial systemic hypertension. On the other hand, his own medical history included nasal plastic surgery and previous neuro-surgical surgery due to recurrent lumbar disc herniation.

A month before admission, he experienced an isolated episode of sharp pain in the right lumbar area, not radiating elsewhere, with vomiting. This episode lasted approximately an hour and resolved spontaneously without any medication. The following month, a similar episode occurred and had a spontaneous regression. On the day of hospitalization, the patient experienced the same painful symptoms, but they were drug-resistant.

On arrival at the ER, tenderness was noted in the left flank, negotiable abdomen, and negative Giordano’s maneuver. The patient’s systemic blood pressure was 160/90 mmHg, without hypotensive treatment. His body temperature was in normal range, except for a single temperature peak of 37.6 degrees Celsius detected on the day after admission to our Nephrology Unit.

At admission, we started intravenous pain medication and conducted instrumental and laboratory tests.

These revealed a normal hemoglobin level, 17 g/dL (normal values: 14–18 g/dL); increased white blood cell count, 14.120 cells/mmc (normal range: 4.30–10.8 cells/mmc); and normal renal laboratory tests, with serum creatinine levels at 1.0 mg/dL (normal range: 0.69–1.30 mg/dL) and BUN levels at 27 mg/dL (normal range: 15–38 mg/dL). The D-dimer value was increased to 884 ng/mL (normal range: 0–500 ng/mL); the homocysteine level was normal at 6.7 mmol/L (n.v.: 5–12 µmol/L); serum creatine kinase was 335 U/L (normal range: 39–308 U/L) and lactic dehydrogenase was 353 U/L (normal range: 87–241 U/L); hepatic transaminases were 65 IU/L (normal range: 13–57 IU/L); and inflammation indices were normal, with a PCR level of 0.29 mg/dL. The standard urine test showed a weak presence of blood cells in the urinary sediment (1–5 Ul, usually absent); lipid levels, total cholesterol, triglycerides, blood proteins, and albumin were all in normal ranges; and the blood bilirubin level was 1.2 mg/dL (normal range: 0.2–1.0 mg/dL).

A nasopharyngeal swab for SARS-CoV-2 detection was negative.

During hospitalization, he had some isolated arterial hypertension peaks but was well oxygenated in ambient air. He continued showing abdominal flank lower back pain in an intermittent mode.

The electrocardiogram results were normal.

The differential diagnostic process was directed toward uncontrolled secondary hypertension vs. abdominal flank pain. Therefore, we performed further laboratory testing for a genito-urinary tract infection or abdominal diseases over an uncontrolled hypertension pattern.

The sampled autoantibodies (ENA, anti-DNA, AMA, and ASTHMA) were negative, while ANA was weakly positive with a “speckled-type” granular pattern. Complements C3 and C4 were within their normal ranges. Thyroid auto-antibodies and thyroid hormone levels were normal. Serum protein electrophoresis showed normal results. The mutational study of the V° Leiden Factor showed a weak alteration in heterozygosity, which was overall considered normal (Mutation-5, 10 MTHFR).

As a clinical instrumental investigation, we performed an abdomen and renal Doppler ultrasound looking for abdominal bowel disease or hydronephrosis, as can be the case with a genito-urinary tract infection. No renal pelvis dilatation or abdomen and major abdominal vessel diseases were observed.

Doppler ultrasound examination of the renal arteries showed a normal profile, such as during the examination of the supra-aortic trunks.

**Figure 1:**
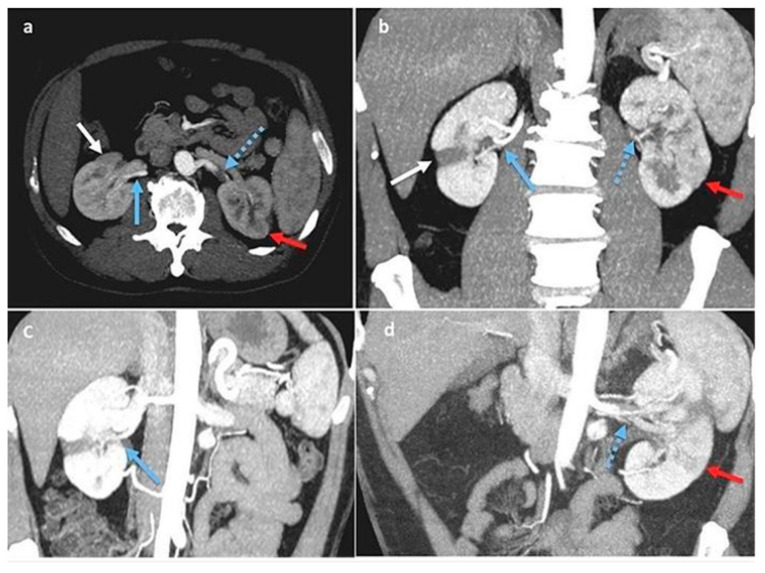
(**a**–**d**): Multiplanar reconstructions on Anglo-CT scan, arterial phase. Right kidney: focal ischemic area (white arrow) associated with sub-acute dissection of the inferior polar artery (blue arrow). Left kidney: acute dissection of renal artery (blue arrow with dots) with focal acute ischemic area (red arrow).

Two days later, a contrast-enhanced complete abdomen computed tomography (CT) scan was conducted [1]. This revealed multiple infarctions and ischemic areas with hypoperfusion in the upper middle third of the left kidney and in a large area of the middle and lower right kidney; the left renal artery exhibited increased intimal thickening and arteritis, similar to thrombotic events, resulting in an extremely narrow residual lumen. The multiplanar reconstruction revealed bilateral renal artery dissection and multiple arterial infarctions disseminated throughout both kidneys (Figure 1a–d).

Aiming for a vessel disease diagnosis, computed arteriography with selective renal and polar artery catheterization [2] was performed as a second-level instrumental exam.

**Figure 2:**
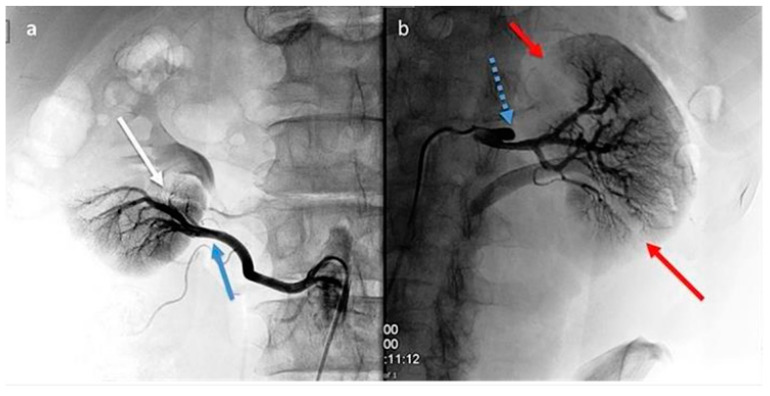
(**a**,**b**): Digital subtraction angiography (DSA). Right kidney (**a**): segmental renal artery occlusion (white arrow) consequent to distal dissection (blue arrows) of the inferior polar artery originating from the abdominal aorta. Left kidney (**b**): renal artery dissection (blue arrow with dots) with occlusion of smaller superior and inferior segmental branches and their corresponding focal de-vascularized areas (red arrows).

This yielded many findings: On the right side, there was an occlusion of a small intraparenchymal terminal branch of the right renal artery, while the inferior polar artery on the right remained patent. On the left side, a dissection of the middle third of the renal artery was observed, characterized by a false occluded lumen and a patent lumen supplying the intrarenal branches; the lower renal polar artery on the left side was also patent (Figure 2a,b).

As an additional abdomen radiological study, we performed magnetic resonance imaging (MRI) of the lower abdominal region (both with and without contrast), which confirmed the presence of multiple triangular areas of cortical ischemia that were more extensive and numerous on the left side. The left ischemic lesions showed slight signs of signal reduction in diffusion-weighted sequences, while no signal reduction spots were observed on the right side (this may indicate variable stenosis and the severity of arterial dissection on both kidneys).

The studies at different imaging levels suggested the recent onset of left kidney lesions, in addition to older lesions in the right kidney. As an anatomical finding, there was a double polar lower renal accessory artery district in both kidneys. The left main renal artery appeared slightly swollen and exhibited reduced opacification, showing thrombotic signs. This anatomical abnormality can also be detected through ultrasound imaging and may be associated with main renal artery dissection in any case.

## 3. Discussion/Conclusions

After we completed the diagnosis, it was necessary to assess the treatment approach for both the short and long term. Therefore, we performed an equipe medical consultation, and no endovascular maneuvers (e.g., endovascular loco-regional fibrinolysis) were necessary for this case [3].

After two days, a partial and transient right flank pain remission radiating to the lumbar area was observed.

The pain recurred suddenly and intermittently but decreased when the patient was in a lying position. Their blood pressure was high during lower back pain episodes, prompting the need for ACE inhibitor therapy to stably reduce it.

According to their medical history, no triggering event (e.g., trauma, excessive sport practice) for artery dissection was detected, and there were no underlying collagenous disease conditions such as Ehlers–Danlos syndrome or fibromuscular dysplasia.

The diagnosis of spontaneous double artery dissection (SRAD) with multiple bilateral kidney infarctions was concluded.

Finally, the patient was discharged to their home and scheduled for a monthly follow-up.

Spontaneous renal artery dissection (SRAD) is a rare clinical event that typically presents with acute low-back or flank pain, hypertension, fever, hematuria, and acute renal failure. The condition is often misdiagnosed or receives a delayed diagnosis due to its relative rarity and non-specific presentation. The diagnosis can be made from contrasted-enhanced computed tomography (CT) scans, and if the diagnosis is positive, vascular multiplanar reconstruction is generally suggested, as it can display lesions more clearly. Over 300 cases have been reported since the first characterization of SRAD; however, to date, a consensus has not been reached on the most appropriate treatment.

Conservative therapy, open surgery, and intravascular intervention have been described as treatments for SRAD.

In this case study, we observed double spontaneous renal artery dissection over a double renal district (lower polar artery in both kidneys).

A pro-coagulation state—as indicated by increased levels of D-dimer and a weak mutation of Factor V of Leiden—could be linked to the multiple causes of renal artery dissection in our case.

The weak positivity of autoimmune antibodies may have played a role in the mixed pathogenesis that could have triggered bilateral renal artery dissection.

In this case, the intermittent and delayed appearance of the symptoms could have been easily misdiagnosed as non-specific abdominal flank pain due to causes such as a genito-urinary tract infection or bowel disease [4]. The recurrence of pain within a short period and its drug resistance were meaningful and necessitated hospitalization.

Uncontrolled blood pressure during an undetermined period (before hospitalization) may have also played a role and, together with a weakly positive autoimmune laboratory pattern, may have contributed to the progress of bilateral renal artery dissection.

The utilized diagnostic algorithm involved ultrasound and renal artery echo-color Doppler as the first clinical approach, which were not highly relevant for the diagnosis. These examinations are typically used to rule out infective genito-urinary diseases, calculi, or gut disease.

As has been reported in many studies, the gold standard for renal artery dissection diagnosis is computed arterial tomography and subsequent arterial selective angiography, providing a fast, non-invasive, and clinically accurate method to identify the site and extension of the dissection.

In our case, we performed computed axial tomography and selective renal artery arteriography, which helped to recognize the double renal artery dissection and multiple bilateral infarctions in the renal parenchymal–arterial district, as well as the double renal lower polar artery.

A clinical approach is required to quickly assess the risk of bleeding and stabilize the dissection to avoid the recurrence of thrombosis and consequent total renal artery lumen occlusion, which could lead to renal infarction [5].

Many authors have stated that endovascular stenting repair is strongly recommended when medical therapy has been ineffective. Some cases have reported successful treatment of SRAD with renal infarction using self-expanding nitinol stents. In our case, this was not necessary due to the size, extension, and gravity of the arterial dissections.

The best treatment for renal artery dissections remains unknown, and there is no shared consensus on the timing of intervention. Revascularization may be evaluated in patients with renovascular hypertension or for improving kidney function (e.g., if impaired renal function after stenosis is reported).

Many other studies have described blood pressure amelioration in patients who underwent endovascular stent placement for uncontrolled hypertension in SRAD.

In our case, we prescribed dual antiplatelet therapy and an ACE inhibitor at discharge.

No lipid-lowering therapy was inducted due to the normal range of lipid levels in the patient.

Remarkably, abdominal lower flank pain can have various causes, and reaching the final diagnosis may be challenging, including searching for genito-urinary tract infections, kidney stones, or bowel disease.

When presented with an adult male aged between 40 and 70 years reporting intermittent (colic-like) drug-resistant flank pain who shows normal renal function but increased white blood cell count, renal artery disease should be considered a possibility.

It is crucial to carry out rapid and accurate diagnosis; so, urgent clinical and instrumental screening approaches such as laboratory tests and computed axial tomography are recommended. Selective renal arteriography is indicated for detailed determination of the renal artery dissection site and type.

Laboratory tests upon admission and investigation of autoimmune antibody panels could be very important, particularly in cases with a known familial history disease or when connective tissue disorders are suspected.

Stabilizing blood pressure must be the primary objective not only during hospitalization but also at discharge when planning clinical and radiological follow-ups for cases that do not require urgent endovascular or open surgery treatment but could worsen or relapse.

In clinical cases, such as our reported one, good clinical practice could involve analyzing previous clinical factors, comorbidities, age class, sex, lifestyle factors (substance abuse, extreme sports, others), and recent strains or trauma before admission that could have led to rapid spontaneous renal artery dissection.

## Data Availability

The original contributions presented in the study are included in the article, further inquiries can be directed to the corresponding author.
